# What to Eat, and What to Drink, and How, and When, and Where to Do It

**Published:** 1869-07

**Authors:** B. H. Pratt


### THE BISTOURY.

ELMIRA, N. Y., JULY, 1869.

**Thad S. UpDeGraff, M. D., Editor.** **Volume V.---No. 2.**

The BISTOURY is published Quarterly, upon the 1st of April, July, October and January, at Fifty Cents a year in advance.

☞ For Club Rates see Prospectus on inside of Title Page.

### What to Eat, and What to Drink, and How, and When, and Where to do it.

BY B. H. PRATT, A. M.

No thoughtful man will deny that Taste, in a state of Nature, is a law unto humanity in the matter of Eating and Drinking; and only a croaker, whom it were base flattery to call an Ass, will claim that Taste, among the civilized, has aught to do with either.

Reason's a fool to Instinct in wise gastronomy.

Culture has spoiled the children of men—perverted their taste as it has their conscience. But this is none of our business. We note the fact—weep becomingly—blame our progenitors—berate the age we live in—tell how *we* would revolutionize the race. This done, we roll our eyes heavenward, and sigh for the depravity of our fellows. How touching.

Still, Dyspepsia is killing this whole generation. Don't believe it? Then let incredulity be thy bliss. Admit it? Come, then, let us reason together.

What shall we eat?

Taste cannot guide—we've trusted to her and fallen. The morsel she calls good under the tongue, is more than likely to be bad in the belly—bad in the blood; to delight, then torture us—to exhilarate, then rack us.

To lay down specific rules—absolute laws for the wholesome guidance to health lost—sure preservers of health possessed; to say to the well, eat and drink thus and live an hundred years—to the ill, eat and drink so, and get vigor, strength and fatness; that were a silly code of a silly giver.

Yet the M. D's all do it.

Do the M. D's practice it? Scarcely.

Given—a list of edibles and potables. Æscuapius will run his finger down the batch and tell you to "eat this—abstain from that—partake sparingly of the other."

What nonsense. As if he who knows (I care not how well) the relations of medication and disease, but knows little of the relations of food to health, and still less of the effect of a certain diet upon me, shall expect my confidence in his ability to say what I shall eat and drink.

Who, then, is the judge?—what the law?

You; no other. Not the Doctor—not even your wise grandmother. Your own feelings are the legislators—common sense the judge—your sense of right the executive. You need no help but your own—foreign aid cannot assist you. You are supposed to be sane. *You* know what hurts you, however much you try to deceive yourself. You know you persist in eating what you ought not, and drinking what you need not; but because Jack and Becky can bolt a huge slice of broiled ham, and swill a big mug of beer apiece at dinner, and feel the better for it, forsooth, these won't hurt you, who after eating a square inch of the former and drinking a swallow of the latter, suffer the pangs of indigestion and remorse for hours.

Stomachs differ. Hence a variety of food has been ordained. It wasn't *all* intended for *you*. Let that alone which evidently wasn't made for you.

Sympathy, for those who cram their stomachs indiscriminately—make animated demijohns of themselves—and then run to a Doctor with "I do wonder what ails me," is thrown away.

What shall you eat? Why, eat everything that *agrees* with you. You'll soon discover that which disagrees—then let it alone, and be honest with yourself. Laws restricting you to certain dishes are unwise and dangerous, unless those laws are the result of your own experience. If mince-pie agree with you, eat it; but be honest with your stomach. If confectionery and after-dinner nonsense inflict no after twinge of conscience nor bowels, continue them; but be honest with your bowels and conscience.

Thus shall we know what to eat.

But what shall we drink?

Can you solemnly affirm that Tea does your organization no harm? Can you place your hand upon your heart and positively assert that Coffee has not harmed you? Can you swear that neither Cider, nor Wine, nor Beer, nor Whisky, nor Brandy, nor the score of compounds known to the caterer in potables—the toddies, slings, juleps, smashes, cocktails, bitters, punches, cobblers, sangarees, tom-and-jerries—are harmful in their effects upon your constitution? Then drink them, one and all. But, my dear fellow, be honest with yourself as you would with your own son or friend, and as you value your precious body and immortal soul.

*How* shall we eat and drink?

Temperately— sufficiently—-composedly—-Physiologically—decently.

Don't stuff yourself. Eat as though you expected to eat again. Know your stomach's ability and respect it. "Verbum sap. sat."

Eat enough. Error upon the side of insufficiency is perhaps as dangerous as upon that of repletion. Abstemiousness is seldom wisdom. The well fed strikes a mean between the ill-fed and over-fed. If you would have an exemplification of the ill effects of *dieting*, (only a human species of starvation) question the face and figure (not the lips) of one of our experimental dyspeptics. You need not go far to find one.

Be calm—jolly if practicable—during your meal. Why so? Consult your fat and jovial friend—you have him now in mind; or better, ask him to dine with you. He will satisfy you that there is nothing like jollity to make the juices of digestion flow.

Physiological eating is the effect of due attention to the proper mastication of food—bringing it to a proper consistency before swallowing—not crowding the organs of deglutition—in a word, not compelling the stomach to do what teeth, lips, tongue and glands ought to do. Give every organ only its own work to do, and rest assured it will do it well.

Eat decently—not as the swine, madly, noisily, slobberingly; but with care, avoiding that which you see unseemly, displeasing, disgusting, in others. This not for health's sake, perhaps, but for decency's sake. It becomes us to be decent in all things.

*When* shall we eat?

There are, who take four regular meals during the twenty-four hours—heaven help 'em! —and munch voraciously between times.

There are, who insanely persist in believing that the animal man gluts itself, and ought to eat but once per day. Such religiously abstain from one bite more than this.

Both Err. To know, is to test both extremes. Ultraisms in regimen meet the same fate with ultraisms in everything else.

But whether we adopt two or three meals per day, (and nature seems to demand one or the other) they should be regular.

Man is a creature of habit, and must yield thereto if he would thrive. The intricate and delicate organism of the human body, like any other delicately and sensitively contrived piece of mechanism, must not be required to do much more nor much less than its quota of work; much less must that work be doubled to-day and halved to-morrow. The successful farmer does not so treat his stock; the iron-founder does not so treat his blazing stacks; the wise eater will not so treat his precious stomach. Whatever hours we set apart for replenishing the body, let us see to it that they be regular even to scrupulousness.

Yet, children have their candy and cake munchings--Sis and Bub their clandestine slice of bread and butter, or secret unripe fruit in the season of it—Youth its confection or saloon tit-bit at any time of day or night—the big grown man his fruit, nuts or what not, when handy. These make colics and spasms and puny forms for the little—diarrheas and cramps and all manner of spells and turns for the big—discomforts for all, save the Doctors. No wonder we are a debilitated race—the wonder is that we are other than crawling, whining invalids. But for this are there Doctors—and Hospitals—and Asylums. Our eleemosynary institutions are prostituted to the relief of self made invalids, whom the world is ignorant enough to pity.

It is in drinking that we chiefly err. The "man of the period" seems a perpetual interrogation which asks "when may we not drink?" It matters little what it be, or where it be; but the when, that's *now* and *ever*. He drinks in company because it's social—alone, because he has no society. He drinks when well, to keep him so—when sick, to make him well. When cold, he drinks to warm him—when warm, to cool him. He drinks when rich because he is rich and can afford it—when poor, because he would forget his poverty. He drinks when jolly, because he's jolly—when blue, because he's blue. Drinks when he wins—drinks when he loses. Men do not write themselves down Asses so often nor so effectually in any other act as in their senseless drinking.

Here I ask pardon of the quadrupedal Ass.

*Where* shall we eat?

At home. Everybody has a home of some sort—mayhap not one in the usual acceptation of the abused word—yet a home. One's hotel or one's attic may be one's home. It's his place to be—his off-duty resort—his home. If one cannot gather about him such company as he likes, he can still make his surroundings homelike. There eat; not in the shop—not at your desk—not at the restaurant, unless absolutely necessary. Eating on the wing is perilous to digestion and demoralizing to good habits. It's unnatural, uncomfortable, boorish, injurious. There's little gain and much loss in the practice, besides, being, in nine cases out of ten, quite unnecessary.

The care of the stomach is no light one. That all disease proceeds directly or indirectly from the stomach, renders the attention to that organ all-important. That which enters in at the mouth cannot be too closely scrutinized. As one evil man may provoke a community to sedition, so one corrupt morsel may cause the corruption of the whole body.

Then let us speedily seek to know better, What to Eat and What to Drink, and How and When and Where to do it.

